# A novel approach to modelling water transport and drug diffusion through the *stratum corneum*

**DOI:** 10.1186/1742-4682-7-33

**Published:** 2010-08-17

**Authors:** Tatiana T Marquez-Lago, Diana M Allen, Jenifer Thewalt

**Affiliations:** 1 Department of Biosystems Science and Engineering, ETH Zurich, Mattenstrasse 26, 4058 Basel, Switzerland; 2 Department of Mathematics, Simon Fraser University, Burnaby, BC, V5A 1S6, Canada; 3 Department of Earth Sciences, Simon Fraser University, Burnaby, BC, V5A 1S6, Canada; 4 Department of Molecular Biology & Biochemistry and Department of Physics, Simon Fraser University, Burnaby, BC, V5A 1S6, Canada

## Abstract

**Background:**

The potential of using skin as an alternative path for systemically administering active drugs has attracted considerable interest, since the creation of novel drugs capable of diffusing through the skin would provide a great step towards easily applicable -and more humane- therapeutic solutions. However, for drugs to be able to diffuse, they necessarily have to cross a permeability barrier: the *stratum corneum *(SC), the uppermost set of skin layers. The precise mechanism by which drugs penetrate the skin is generally thought to be diffusion of molecules through this set of layers following a "tortuous pathway" around corneocytes, i.e. impermeable dead cells.

**Results:**

In this work, we simulate water transport and drug diffusion using a three-dimensional porous media model. Our numerical simulations show that diffusion takes place through the SC regardless of the direction and magnitude of the fluid pressure gradient, while the magnitude of the concentrations calculated are consistent with experimental studies.

**Conclusions:**

Our results support the possibility for designing arbitrary drugs capable of diffusing through the skin, the time-delivery of which is solely restricted by their diffusion and solubility properties.

## Introduction

Recently, the potential for using skin as an alternative path for administering systemically active drugs has attracted considerable interest [1]. Among some of the most active topics of research is the study of the physical properties of the Stratum Corneum (*SC*), which constitutes the uppermost set of skin layers. The *SC*'s main function is providing a barrier against the loss of physiologically essential substances, and to the diffusion of potentially toxic chemicals from the external environment into the body. It also constitutes a protection against mechanical insults and is the primary defence against ultraviolet light; screening out more than 80 percent of incident irradiation.

**Figure 1:**
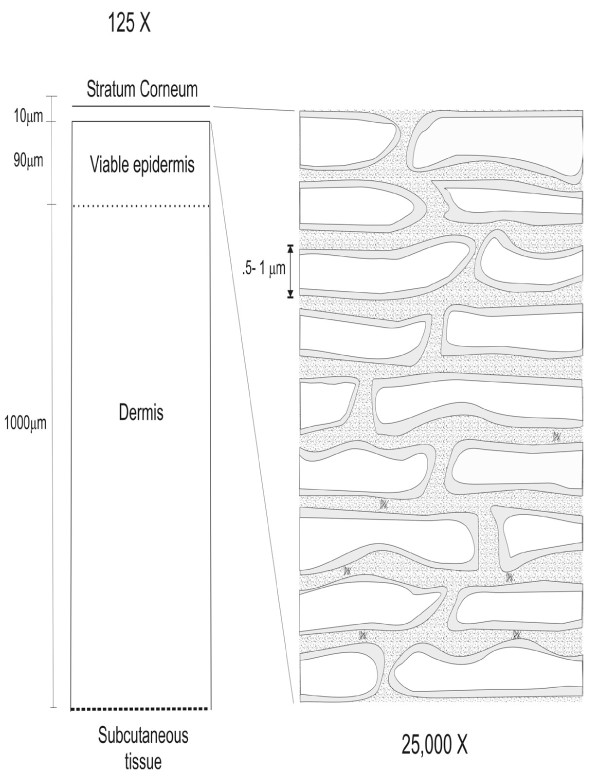
**Structure of the s*tratum corneum***.

The *SC *is the uppermost layer of the epidermis (see Figure 1) and consists of a network of cells called corneocytes, embedded in a lipid matrix. This structure between the cells of the SC is quite unique in mammalian membrane biology, and has been long considered a "solid lipid crystal" [2]. Beneath the *SC*, the viable epidermis is chiefly composed of specialized cells known as keratinocytes [3]. These keratinocytes grow in size and remodel their cytoplasm, preparing to transform into corneocytes through a process of terminal differentiation followed by programmed cell death. The corneocytes, although devoid of a metabolism, confer most of the skin resistance to chemical and physical attacks, and in their normally dehydrated state also provide obstacles against water loss through the skin.

**Figure 2:**
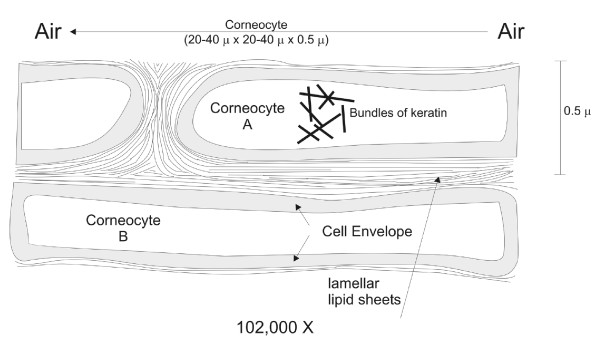
**Dimensions of the corneocytes and intercellular channels**.

A diffusing molecule has to cross multiple bilayers before it either encounters viable tissue, where it is required to act locally, or blood supply if it is to act systemically [4]. Hence, the precise mechanism by which drugs penetrate the skin is generally thought to be the diffusion of molecules through the *SC *following a tortuous pathway around dead cells [4]. Namely, the *SC *is composed of stacked, polyhedral corneocytes surrounded by lipid membranes and, for the sake of simplicity, can be thought of as a brick wall composed of dead cells (the "bricks") and intercellular lamellar membranes (the "mortar"), as depicted in Figure 2. Such geometry has been previously considered in simplified one-dimensional [5] or two-dimensional diffusion models [6-8], and within studies linking permeability and solubility [6], SC geometry and permeability [9], and the dependence of diffusivity and general SC barrier properties on permeability, corneocyte alignment and lipid content [6,9-11].

Skin has previously been considered a porous medium [12] but, to the best of our knowledge, fluid flow models in generalized porous media had not been previously applied to the SC. In this paper, we summarize our results in [13], where a simple three-dimensional porous media model for water transport and drug diffusion in the SC was presented, much as it is used for the conceptually similar problem of groundwater flow and contaminant transport. A simplified version of the general problem of water flow through a porous medium is formulated. Also, the general problem of mass transport within a porous medium is considered; the logical application being the analysis of drugs being applied to the *stratum corneum *(*SC*).

In our approach, the *SC *is considered to be composed of a periodic structure of cellular skeletons, separated by intercellular lipid channels. The specific biological data that are used to define the parameters for the model and guide the simulations are used in a simplified context. Variables that increase the complexity of the model, such as temperature, or variables that arise from the introduction of chemical drugs that would alter its physical structure are generally excluded.

It should be noted that the impermeability of corneocytes can be controversial, since corneocytes can swell after prolonged soaking in excess water. However, swelling does not hint to any permeability property, but simply means that corneocytes contain proteins that can bind water molecules. Moreover, corneocytes are surrounded by a tough cellular envelope consisting of covalently bound proteins and lipids, vastly differing from mammalian plasma membranes, which are quite permeable to water. Hence, corneocytes are largely considered to be impermeable, while lipids provide the major transdermal route for water transport (cf. [14], and references therein).

Our approach for modelling the diffusion of water through skin layers involves the concepts of flow through porous media as applied to groundwater flow modelling [15,16]. Following such an approach, geologic materials -and in our case the SC- may be considered "equivalent porous media", which means that the pores (voids) and grains comprising the matrix are treated as a continuum, and equivalent macroscopic hydraulic properties are assigned to it. Mass transport in water occurs via the processes of advection (including mechanical dispersion) and diffusion. Advection is mass transport due to water flow, in which the mass is dissolved; thus, mass will follow the fluid's streamlines. Dispersion causes a zone of mixing to develop between adjacent fluids of different compositions, and thus, will spread mass beyond the region it normally would occupy due to advection alone. In three dimensions, dispersion spreads mass in transverse horizontal and vertical directions, as well as longitudinally. Diffusion occurs along a concentration gradient, and may transport mass in a direction opposite to the fluid flow if concentration gradients are high enough or flow velocities are low. In such cases, advection and diffusion are competing processes.

A final consideration is the boundary conditions for both water flow and mass transport. One serious obstacle for building an accurate model within the *SC *is the unreliability and general lack of experimental data; parameters such as pressure (for boundary conditions) are either unknown or vaguely referenced. In our approach, the interpretation of the boundary conditions for water flow is quite straightforward, and involves specifying the head or pressure along the boundary, a specified flux across the boundary, or a combination of the two, in which the flux rate is variable with the head difference across the boundary. In the case of mass transport, boundary conditions include a specified concentration along the boundary, a concentration diffusive flux across the boundary, or a combination of the two, which could represent a constant chemical flux with a specified concentration or pulse-type loading with constant input fluxes, to name a few.

As is the case in all predictive models, several simplifications and assumptions have been made to describe the SC. However, no model exists without simplifications, and modellers must find a balance between an adequate representation of the physical phenomena (which may vary, depending on the focus of the study) and the associated computational costs to solve the model. In this sense, if a simple model predicts behaviour as demonstrated by a real system, the simplifying assumptions are duly justified. Henceforth, a model can be tuned, and the importance of various factors can be better estimated.

Our proposed model does not claim to be an all-encompassing view of the structure and functionality of the SC, but aims to provide for a basic ground upon which refined models in a variety of three-dimensional skin architectures and conditions could be simulated. For instance, by extending to a porous media context new and previous considerations on corneocytes size and arrangement [7,8,11], permeability changes [6,9], and variation of intercellular lipids [10], to name a few. As such, a wide variety of physiological and pathological conditions can be analyzed, in a more systematic and practical way.

## Theory

### Geometry of the SC

The dimensions of both corneocytes and intercellular channels vary over a wide range, depending on the hydration level, the part of the body, and the organism from where a sample is extracted. Typical corneocyte dimensions can vary from 10 *μ*m × 10 *μ*m × 0.5 *μ*m to 40 *μ*m × 40 *μ*m × 1 *μ*m; the "prototypical" one having dimensions of 20 *μ*m × 20 *μ*m × (0.5-1.0) *μ*m. Moreover, the width of the intercellular channels usually lies in the range of 0.2-1.0 *μ*m between adjacent corneocytes (N. Kitson, personal communication, and [17-20]).

The matrix that comprises the material present in the intercellular channels is composed of hydrophobic lipids arranged in multiple lamellar sheets, along with remnants of intercellular attachment plaques [21]. The important feature of all these lipids is that they structure themselves into fairly solid, ordered arrays [22]. Thus, the lamellar membranes of the *SC *provide the barrier to systemic water loss and impede the uptake of xenobiotics.

### *Stratum Corneum *as a Porous Medium

The pathways for passive diffusion across mammalian epidermia are thought to be: (1) between the cells of the *SC *(intercellular pathway), (2) through the cells of the *SC*, and (3) through appendages such as hair follicles and sweat glands. Although the relative importance of each is still not clear, there is a general consensus that the intercellular pathway plays a major role in water and small molecule transport.

Since the *SC *is obviously a heterogeneous material, representing it by a porous medium model would, in the simplest case, divide the *SC *into two domains: the pore space and the impermeable space, distinguished by their distinct conductive properties. Rigorously, the physical counterparts of both domains would also be heterogeneous. In particular, the passive diffusion of molecules across the *SC *via the intercellular pathway can be described in terms of diffusion within a single lipid layer, diffusion perpendicular to the lipid lamellae, and interlamellar diffusion. So, for example, water diffusion across an array of stacked lipid layers having coexistent liquid crystalline (fluid) and crystalline regions, the membrane component of the diffusion path can itself be imagined as a porous medium; the solid lipids being the impermeable space and the fluid lipids composing the pore space.

For the sake of simplicity, we consider the intercellular channels to be homogeneous and isotropic with a significantly higher permeability than the corneocytes. Thus, in our simplified model, molecules should follow a tortuous path around corneocytes, and their pathlines would depict a "direct" route going from higher to lower hydraulic pressure, without explicitly taking into consideration the nano-scale tortuosity of the lipid lamellae that form the intercellular channels. Nevertheless, this nano-scale tortuosity will be partially accounted for in the average characteristics of the 'pore space', their effects not being altogether neglected. Alternatively, one could explicitly account for anisotropies, at the expense of exceedingly large computation costs, a numerical topic that lies outside the scope of this study.

### Modelling flow in porous media

Following the methodology described in [15,16], we will develop the partial differential equation describing flow in a porous medium, based on the law of conservation of mass, particularly applied to a unit representative elementary volume (REV) with dimensions Δ*x*, Δ*y*, Δ*z*. This PDE refers to time variations in the hydraulic head, expressed in units of height. The hydraulic head *h *= *h(x, y, z, t) *is equivalent to the sum of the elevation head *z *and the pressure head (*P*/*ρ*_*ω*_*g*) where P is the pressure, *ρ*_*ω *_is the density of the fluid, and *g *is the gravitational acceleration. In fluid dynamics, head is a concept that relates the energy in an incompressible fluid to the height of an equivalent static column of that fluid. From Bernoulli's Principle, the total energy at a given point in a fluid is the energy associated with the movement of the fluid, plus energy from pressure in the fluid, plus energy from the height of the fluid relative to an arbitrary datum. In a porous medium, the velocity is small enough that the energy associated with movement of the fluid is negligible.

A macroscopic constitutive equation (referred to as Darcy's law) relates the hydraulic gradient to the mass (fluid) flux in three dimensions using constants of proportionality (*K*_*x*_, *K*_*y*_, *K*_*z*_). For instance, in one dimension the fluid flux is defined by:

$$fluid\;flux=K\frac{dh}{dx}.$$

The hydraulic conductivity, *K*, is a property of both the porous medium and the fluid, and is defined by:

$$K=k\frac{\rho_{w}g}{\mu }$$

where *k *is the intrinsic permeability of the porous medium (in units of squared length) and *μ *is its viscosity. *K *can be anisotropic in each of the three coordinate directions such that the principal directions of anisotropy are not equal (*K*_*x *_≠ *K*_*y *_≠ *K*_*z *_). As well, *K*_*x *_, *K*_*y *_, *K*_*z *_are typically assumed to be collinear to the *x*, *y*, *z *axes. If it is not possible to align the principal directions of anisotropy with a rectilinear coordinate system, one should necessarily consider all the components of the tensor:

$$K=\left[\begin{array}{ccc} K_{xx} & K_{xy} & K_{xz}\\ K_{yx} & K_{yy} & K_{yz}\\ K_{zx} & K_{zy} & K_{zz} \end{array}\right].$$

Considering the mass flux of fluid into an REV, and by further defining R* Δ*x*Δ*y*Δ*z *to be the volumetric inflow rate (with R* > 0 referring to a source of water, and R* < 0 to a sink, the transient flow can be described

$$\frac{\partial }{\partial x}\left(K_{x}\frac{\partial h}{\partial x}\right)+\frac{\partial }{\partial y}\left(K_{y}\frac{\partial h}{\partial y}\right)+\frac{\partial }{\partial z}\left(K_{z}\frac{\partial h}{\partial z}\right)=S_{s}\frac{\partial h}{\partial t}-R^{*}$$

where *S*_*s *_is the specific storage, defined as the volume of water released from storage per unit change in head *h*, per unit volume of saturated porous medium. The specific storage of a porous medium can be described by:

$$S_{s}=\rho_{\omega }g\left(\beta_{p}+n\beta_{\omega }\right)$$

where *β*_*p *_is the vertical compressibility of the porous medium, and *β*_*ω *_is the fluid compressibility. Hence, for isotropic conditions (*K*_*x *_= *K*_*y *_= *K*_*z*_), the equation that describes flow in a saturated porous medium is:

$$\nabla^{2}\text{h}=\frac{\rho_{\omega }g(\beta_{p}+n\beta_{\omega })}{\text{K}}\cdot \frac{\partial \text{h}}{\partial \text{t}}=\frac{S_{s}}{K}\frac{\partial h}{\partial t}$$

and if one intends to model steady state flow, the steady-state Laplace equation is obtained. If the porous medium is unsaturated, whereby the pore spaces are only partially filled with water, *K *becomes a function of the pressure head, and consequently, of the hydraulic head. Hence, the steady-state non-linear unsaturated anisotropic flow equation is defined as:

$$\frac{\partial }{\partial x}\left(K_{x}h\frac{\partial h}{\partial x}\right)+\frac{\partial }{\partial y}\left(K_{y}h\frac{\partial h}{\partial y}\right)+\frac{\partial }{\partial z}\left(K_{z}h\frac{\partial h}{\partial z}\right)=0.$$

Thus, to solve this equation for steady state flow conditions, information on the hydraulic conductivity (permeability) of the different components of the *SC *and how the pressure varies within each is needed. For transient flow simulations, information on the rheological properties is needed, in order to define the specific storage coefficient. Finally, in this formulation, the density and viscosity of the fluid are assumed to be constant, as well as its temperature.

### Modelling mass transport: advection and dispersion

In order to simulate mass transport in water, one has to consider advection - the mass following the fluid's streamlines - along with dispersion or mixing effects, and simple diffusion. In applications with significant fluid movement, scale-dependent mechanical dispersion is usually the more important factor. In slow moving fluids or over long time periods, diffusion can become more important.

The process of dispersion causes a zone of mixing to develop between fluids of distinct compositions, and will spread mass beyond the region it normally would occupy due to advection alone. It should be noted that the concept of dispersion used here differs from that of common Applied Mathematics usage, where dispersion is the dependence of phase velocity on wavenumber. If dispersion is to be incorporated in the advection-diffusion equation, it should be reflected in the velocity term.

In a simple non-porous system, Fick's law describes the chemical mass flux to be proportional to the gradient in concentration. For instance, in one dimension, this results in:

$$F_{x}=-D_{d}\frac{dC}{dx}$$

where *F*_*x *_is the mass flux (of the drug in this case) in the x-direction, C is the concentration, and *D*_*d *_is the molecular diffusion coefficient. In the case of mass transport in porous media, an effective diffusion coefficient is defined to take into account the tortuosity and effective porosity of the porous media.

Advective transport occurs simply due to the moving fluid as defined by:

$$F_{x}=v_{x}\cdot n_{P}\cdot C$$

where *υ*_*x *_is the average linear velocity vector along the x direction, and *n*_*p *_is the effective porosity of the porous medium (often lower than the total porosity). The average linear velocity is then defined as the ratio of the fluid flux to the effective porosity.

Dispersivity is a property of the porous media and has been shown to be scale dependent. Mechanical dispersion can occur in the same direction as the flow, but also in directions perpendicular to the flow (laterally and vertically). Dispersion in all directions, however, is dependent on the average linear velocity in the direction of fluid flow. Therefore, dispersion is linked to the velocity via the dispersivity values in each of the longitudinal (*α*_*L*_), transverse horizontal (*α*_*TH*_) and transverse vertical directions (*α*_*TV*_) through a series of equations (see [15]). 

By further considering a hydrodynamic dispersion coefficient *D *(that incorporates the combined effects of diffusion and mechanical dispersion), and all three dimensions of the problem, one can construct an appropriate advection-dispersion equation. If it is the case that the dispersion coefficient is constant, this equation would simply be:

$$\frac{\partial \text{C}}{\partial \text{t}}=D\nabla^{2}\text{C}-\upsilon \cdot \nabla \text{C}+\text{C}\nabla \cdot \upsilon$$

where *C *still denotes the concentration (for instance, of a drug or pollutant), *D *is the hydrodynamic dispersion coefficient, and *υ *is the average linear velocity vector with components *υ*_*x*_, *υ*_*y*_, *υ*_*z*_. Under zero flow conditions, *υ *would become zero and only diffusion would be active. The justification for treating dispersion in this manner is purely a practical one, and stems from the fact that the macroscopic outcome is the same for both diffusion and mechanical dispersion. The actual physical processes, however, are entirely different. For a more detailed derivation of the flow and advection-dispersion equations, please refer to [13].

### Pressure within *Stratum Corneum*

There is a lack of available data for defining pressure across the SC. Despite a thorough literature search, no specific measurements were found, and thus, many types of pressure had to be considered. In the porous media context, capillary pressure is a basic parameter for studying the behaviour of two or more immiscible fluid phases. The height of capillary rise can be defined by:

$$h_{c}=\frac{P_{w}}{\rho_{\omega }g}$$

where *h*_*c *_is the capillary rise and *P*_*w *_is the capillary pressure between water and air in the porous medium. Thus, we refer to "capillary head", and the difference between the head pressures at the top and bottom layers of corneocytes would define the pressure gradient. Other types of pressure worth considering are: hydrostatic blood pressure, hydrostatic capillary pressure [16], gauge pressure, vapour pressure, osmotic pressure, and tissue (interstitial) hydrostatic pressure. In any case, the pressure gradient within *SC *is thought to be outward-oriented.

In the case of skin as a whole, it has been shown that the spatial and temporal profiles of pressure, stress and fluid velocity depend on the permeability, overall fluid drainage and elasticity of the tissue [23]. Thus, tissue is considered to be a fluid-filled, porous, elastic material. The fluid and solid phases are each inherently incompressible, and tissue deformation is described as a change in the relative fluid volume. The two phases are assumed to exist in mechanical equilibrium, and a generalized Darcy's law characterizes interstitial fluid movement [23]. From here, it is viable to consider such ideas to be applicable to the case of *SC*.

### Permeability of the *Stratum Corneum*

Although many results have been reported for the permeability coefficients of the lipids within the *SC *[24,25], for simplicity we consider the permeability coefficient within the intercellular channels of mammalian *SC *to be in the order of 10^-3 ^cm/sec (or 10 *μ*m/sec) [26], (corresponding to a temperature range of 25°C to 28°C), while corneocytes are considered to be impermeable (N. Kitson, personal communication, and [14]).

### Rheological Properties of the *Stratum Corneum*

The elastic properties of the *SC*, measured by the elastic modulus, should be taken into consideration if a transient simulation of the main equation of flow through a porous medium is intended. First, the elastic modulus should be translated into the bulk modulus, which can be easily achieved through the use of Poisson's ratio. This quantity has been measured for a large number of materials. Unfortunately, no ratios were found for the *SC *despite a thorough literature search, nor were the values found of volumetric stress and strain, from which the bulk modulus can be directly derived. This is the main reason why transient fluid flow simulations are not performed in this work (a steady pressure gradient was assumed). Explanations [13] of some of the rheological properties of the *SC *should allow for future consideration, while it is worth noticing simpler diffusion models (for instance [27]) have attempted to describe transient behaviour without pressure, nor elasticity.

## Model Construction and Computational Method

**Figure 3:**
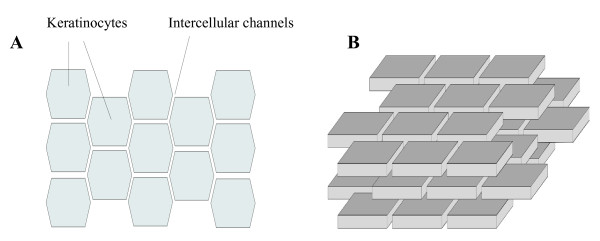
**Brick and mortar structure of corenocytes**. (a) View from above and (b) three-dimensional lateral view.

Physically, the corneocytes resemble three dimensional bodies with hexagons on the top and bottom faces (Figure 3a). For simplicity, we consider them to be thin three-dimensional bodies with rectangles in both faces (Figure 3b). Such simplified corneocytes will be ordered in stacked layers, resembling a "brick and mortar" structure.

Except for very simple systems, analytical solutions of the main equation of flow through a porous medium are rarely possible. Thus, a numerical approach was considered. Here, we shall denote the simulation domain by Ω, and its boundary by Γ (where Γ_T _constitutes the top boundary of the SC, and Γ_B _the bottom boundary). Our simulations will refer to the solution of two separate problems. The first is the flow problem, denoted by:

$$\begin{array}{c} \frac{\partial }{\partial x}\left(K_{x}\frac{\partial h}{\partial x}\right)+\frac{\partial }{\partial y}\left(K_{y}\frac{\partial h}{\partial y}\right)+\frac{\partial }{\partial z}\left(K_{z}\frac{\partial h}{\partial z}\right)=0\;in\;\Omega ,\\ \frac{\partial h}{\partial \vec{n}}=0\;in\;\Gamma \backslash \{\Gamma_{\text{T}},\Gamma_{\text{B}}\},\\ \text{h}(\Gamma_{\text{T}})={\text{h}}_{0}\text{ and h}(\Gamma_{\text{B}})={\text{h}}_{1}. \end{array}$$

As *h*_*1 *_>*h*_*0*_, fluid flow is directed upward. The equation is solved for *h *= *h(x, y, z) *under steady state conditions. Once a pressure profile has been obtained, the average linear velocity vector *υ *is determined spatially throughout the domain, and is then used to solve the drug transport problem, denoted by:

$$\begin{array}{c} \frac{\partial \text{C}}{\partial \text{t}}=D\nabla^{2}\text{C}-\upsilon \cdot \nabla \text{C}+\text{C}\nabla \cdot \upsilon ,\\ \text{C}(\Gamma_{\text{T}1})={\text{C}}_{0}\forall \text{t}, \end{array}$$

where Γ_T1 _denotes a maximal square source area surrounded by an area of zero concentration (see Mass transport simulations section), *C *= *C*(*x, y, z, t*) is the concentration of an arbitrary drug, and the coefficient *D *represents the hydrodynamic dispersion coefficient (accounting for both the effective porosity and tortuosity of the porous medium).

To solve problems using our modelled structure of the physical domain, we rely on the use of numerical techniques. We assume that our problem solution can be considered to be periodic (cfr. Appendix), so we construct a three-dimensional computational cell for which the steady state flow (without any additional sources of water) and transient solute transport (without chemical reaction) are to be calculated. The discretization of the equations for groundwater flow modelling is not trivial, largely due to the fact that the *SC *is a heterogeneous medium. Thus, a precompiled software package, Visual MODFLOW [28] with MT3D (Waterloo Hydrogeologic Inc., 2000), was used. This software is commonly used for modelling groundwater flow and chemical transport through porous media, providing a solution for the fluid flow equation and the advection-dispersion equation within a discretized domain using the method of finite differences.

### Grid Construction

**Figure 4:**
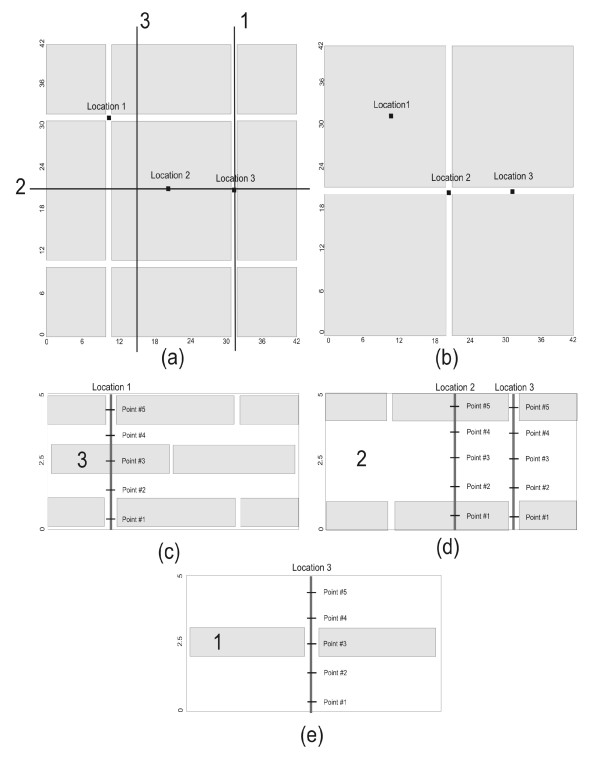
**Layers of corneocytes**. (a) plan view of first and third layers. (b) plan view of second layer. (c) (d) and (e) Cross-sections corresponding to lines 3, 2 and 1, respectively as indicated on (a). Location of concentration profiles and observation points for each depth indicated. Grid dimensions in metres for the scaled domain (5 m = 5 *μ*m).

In consideration of the "brick and mortar" structure, we discretized a basic computational cell composed of several layers of corneocytes (3 in our model), separated by their corresponding intercellular channel layers. In principle, the basic computational cell is regarded as representative of a periodic behaviour. Within the intercellular subdomain, the permeability is of the order of 10 *μ*m/sec [24], while corneocytes are considered practically impermeable [20]. Plan views of model layers are depicted in (a) and (b) in Figure 4. The first and third layers are represented by (a), whereas (b) represents the second (middle) layer. This basic cell is used for all simulations, and it is worth noting that this is only one out of many possible periodic and symmetric configurations. Cross-sectional views for the section lines 1- 3 are shown in (c-e), respectively.

For our basic computational cell, every corneocyte was considered to be a regular three-dimensional box with dimensions 20 *μ*m × 20 *μ*m × 1 *μ*m and each layer was designed to contain at most four whole corneocytes in any horizontal plane and three in a vertical plane. Intercellular channels similarly were assigned a thickness of 1 *μ*m. Because Visual MODFLOW can only process information in the dimensions of metres or inches, it was necessary to scale all the dimensions and hydraulic properties of our problem by a factor of 10^6^, translating the model dimensions from micrometres to metres. Thus, each corneocyte was assigned a size of 20 m × 20 m × 1 m and the intercellular channels were 1 m thick, for a total model domain size of 42 m × 42 m × 5 m (3 corneocytes separated by 2 intercellular channels). Nevertheless, the reader should bear in mind that such scaling does not affect the solution, qualitatively nor quantitatively, since all parameters were scaled accordingly.

To control the error, a uniform discretization was initially implemented using 168 rows and columns, and 20 layers dividing the vertical plane. Next, we used a "telescopic mesh refinement" technique, ending up with 24 rows and columns within each intercellular channel region and 8 within each corneocyte region (preserving the number of layers).

### Permeability, Porosity and Flow Boundary Conditions

As previously mentioned, the size of the corneocytes has been scaled in the model, and to preserve qualitative and quantitative behaviour it is necessary to do the same for all other parameters considered in the simulations. The permeability model for input to MODFLOW consisted of a permeability of 10 *μ*m/sec within the intercellular channels, which was scaled to 10 m/sec in the model. The corneocytes were initially assigned a permeability of 10^-6 ^*μ*m/sec (or 10^-6 ^m/sec in the scaled model). It is worth noting, however, that through a sensitivity analysis we determined that the pressure distribution remained the same regardless of whether a value of 10^-6 ^m/sec or 10^-4 ^m/sec was used. Thus, a difference in permeability of at least four orders of magnitude is sufficient to reproduce the desired qualitative behaviour. Moreover, an effective porosity of 0.27 was assumed to represent the *SC *[29].

Zero flux boundary conditions were used for all lateral faces of the computational cell, based on the fact that the behaviour in the horizontal direction is expected to be periodic. As mentioned previously, exact measurements of the pressure gradient across the *SC *are not reported in the literature. The top and bottom faces had to be set with appropriate values in order to define the pressure gradient. In consideration of the boundary conditions for the top and bottom faces, atmospheric pressure is equivalent to 760 mmHg (or 101,325 N/m^2^) and the density of water is approximately 1.0018 × 10^3 ^kg/m^3 ^at 20°C. Thus, the head pressure at the top of the *SC *is approximately 10.3137 × 10^6 ^*μ*m, which we shall consider as 10 × 10^6 ^*μ*m.

If we choose a pressure 0.33 mmHg higher than the atmospheric pressure to be found at the bottom of the *SC*, this would translate into an approximate difference of 4,000 *μ*m in head values, over roughly a 10 *μ*m thickness (or, equivalently, 2,000 *μ*m over the model thickness of 5 *μ*m). These would correspond to a specified head pressure boundary condition of 10 × 10^6 ^*μ*m across the top layer and a specified head pressure boundary condition of 10.002 × 10^6 ^*μ*m across the bottom layer. This gradient by far dominates any possible baseline physiological fluctuations in pressure, and provides an upper bound for our numerical simulations.

Now, due to the uncertainty in the estimates of pressure gradient, we undertook simulations for two different pressure differences; one set at the values above (a total difference of 2,000 *μ*m), and a second set at a much lower pressure gradient (three orders of magnitude lower, namely 2 *μ*m difference in pressure). The lower pressure difference corresponds to 10 × 10^3 ^m and 10.002 × 10^3 ^m applied to the top and bottom boundaries, respectively. These two simulations were done in order to examine the effect of pressure gradient on the results. For the simulations undertaken at the higher pressure difference, we found that occasional numerical instabilities in the mass transport simulation resulted due to the excessive high gradient (data not shown). This is because a 2,000 m head difference was applied across a 5 m problem domain. Nonetheless, a set of solutions was obtained (see [13]), and the simulations presented here were carried out with the lower pressure gradient, the results of which are further discussed in the results section.

### Dispersivities, Diffusivity and Concentration Boundary Conditions

Longitudinal dispersivity was assigned a scaled value of 0.5 m, whereas horizontal transverse and vertical transverse dispersivities were assigned scaled values of 0.05 and 0.005 m, respectively. Here, one should note that transverse dispersivities are significantly smaller than the longitudinal dispersivity for the skin [20].

Some experiments show that the average diffusion coefficient for water within the *SC *is 3.8 ± 1.3 × 10^-9 ^cm^2^/sec [29,30], a physiological saline infusion called FITC-conjugated dextran has a diffusion coefficient of 10^-7 ^cm^2^/sec within the *SC *[29], and sodium dissolved in water diffuses at a rate of 13.3 × 10^-6 ^cm^2^/sec in many materials [16]. For the simulations, we consider an effective diffusion coefficient of 10^-7 ^cm^2^/sec, equivalent to 10 *μ*m^2^/sec (or 10 m^2^/sec in the scaled model domain).

We simulated the application of a transdermal drug (source) to the skin over a long or continuous time interval; a constant source concentration boundary condition was applied to the model domain surface (i.e., the outer surface of the *SC*). This type of boundary condition effectively holds the concentration at the model surface constant in time. A specified concentration of 10 mg/L was used, and the condition was implemented over a square source area surrounded by a border of zero concentration on the top surface particularly.

### Mass Transport Simulations

Mass transport simulations were done using MT3D (Mass Transport in 3D), a separate code that is contained within Visual MODFLOW, which solves the advection-dispersion (including diffusion) equation at either steady-state or as a function of time. All model runs for mass transport were done in transient time, using the pre-determined pressure field at steady-state. This software automatically implements the time step that complies with its stability criteria, the related number of transport steps that should be used for a simulation. The total simulation time was set to 1 sec with the maximum number of time steps equal to 1000. These parameters were sufficient for convergence of the solution. Simulation results were saved every 5 transport steps.

It should be noted that the reason behind considering a square source area surrounded by an area of zero concentration, Γ_T1_, is purely numerical. Namely, the package MT3D in Visual MODFLOW requires at least one boundary condition to be specified, which we prescribed at the top layer, alongside initial conditions. However, both conditions considering the full top layer could not be simultaneously accounted for by the software. Hence, we did not consider the three first and last columns and rows of the top layer as concentration sources for the calculation of concentration profiles.

### Concentration Observation Points

For the mass transport problem, multiple concentration observation points were introduced into the model; five depths at each of three profile locations as shown in Figure 4. Such points were chosen to be at locations where the ray that defines them passes through at least one vertical layer of intercellular channel. The discrete locations used for concentration measurements were at 0.5, 1.5, 2.5, 3.5 and 4.5 m depth, where the origin of the model was the bottom left. The points are labelled 5 through 1, from the top down at each profile location (see Figure 4).

Location #1 allows us to obtain the time evolution of concentration for observation points within an intercellular channel, followed by a corneocyte, followed by a second intercellular channel. Correspondingly, location #2 refers to points within a corneocyte, followed by an intercellular channel, followed by a second corneocyte. Observation points located in intercellular channels only are to be found at location #3.

The concentration of the chemical was reported as a function of time at each observation point. The resulting plot of concentration versus time is referred to as a "breakthrough curve".

### Solvers

We used the WHS (Waterloo Hydrogeologic Solver) solver for obtaining the flow solution in Visual MODFLOW. This solver uses a Bi-Conjugate Gradient Stabilized (Bi-CGSTAB) acceleration routine implemented with Stone incomplete decomposition for preconditioning of the groundwater flow partial differential equations. The solver will approach the solution iteratively, using the specified domain discretization.

To solve the advective component of transport within MT3D, we used the Modified Method of Characteristics (MMOC), while the advection-dispersion equation was solved using a modified incomplete Cholesky decomposition. Both the relative convergence criterion and the minimum saturated thickness as a fraction of cell thickness were set as the code default values (10^-4 ^and 10^-2 ^units, respectively).

## Results

### Pressure Distribution

**Figure 5:**
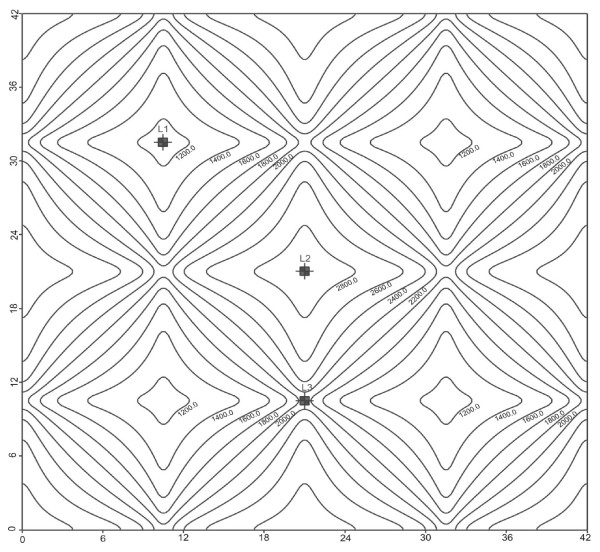
**Plan view of head (pressure) contours within a 42 *μ*m × 42 *μ*m layer composed of intercellular channel lipids only, as obtained through MODFLOW simulation**. Locations (L1, L2 and L3) of monitoring profiles shown.

**Figure 6:**
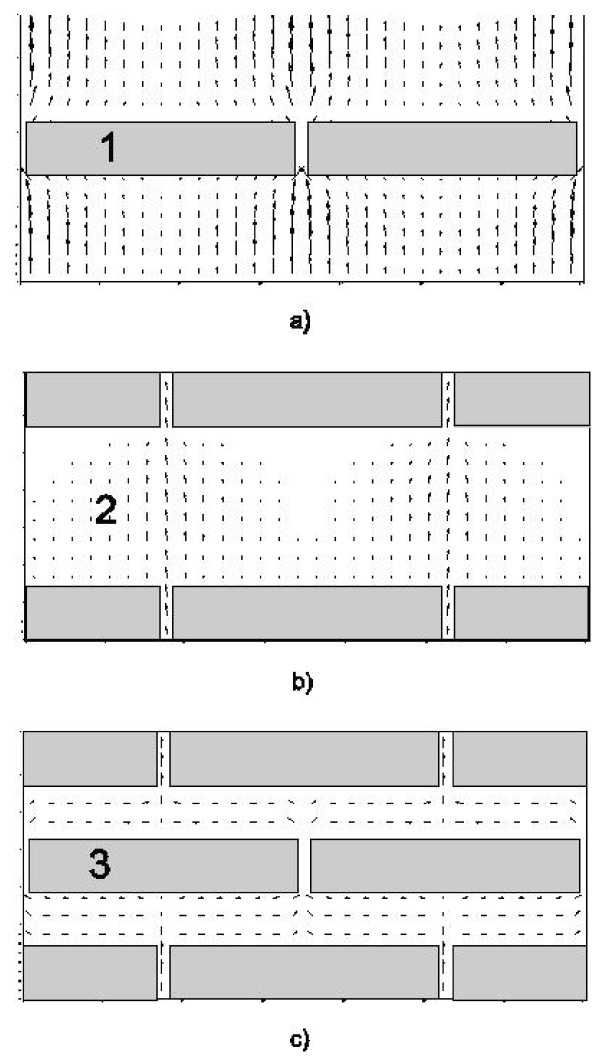
**Velocity vectors for each 42 *μ*m × 5 *μ*m cross-section (1, 2, 3) around corneocytes and through intercellular channels**. Vectors are scaled. Section locations 1, 2, and 3 shown in Figure 4.

The permeability model results in channelized flow as anticipated. Pressure head distributions result in the fluid following a tortuous path through the intercellular channels and around the corneocytes. Due to the latter, we focused in intercellular pressure distribution profiles. Numerical contours are shown in Figure 5, while scaled velocity vectors (which highlight the tortuosity of the flow) for each cross-section are illustrated in Figure 6.

**Figure 7:**
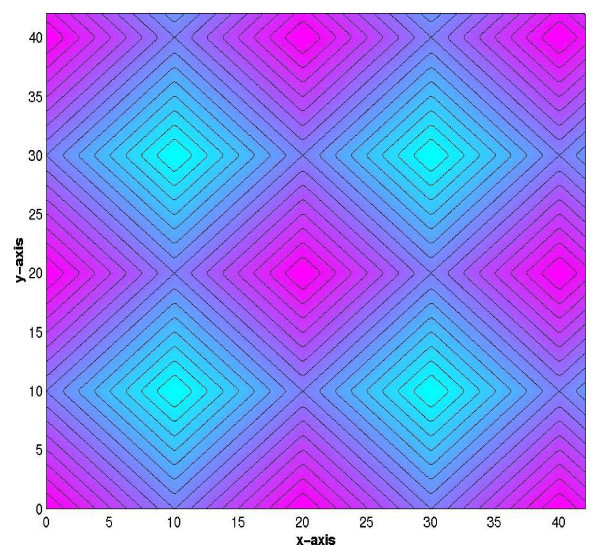
**Analytic solution of pressure distribution within a 42 *μ*m × 42 *μ*m layer composed of intercellular channels, as obtained using Green's functions and the method of images**.

Given that pressure profiles are used subsequently in the mass transport simulations, we benchmarked our pressure results with an analytical model (Figure 7), based on Green's functions and the method of images (for details please refer to the Appendix). From these results, we can argue that both our scaling and Visual MODFLOW yield solutions to the flow problem as would be theoretically expected.

### Drug Diffusion Results

Following the simulations for fluid flow to establish the pressure field, a series of mass transport simulations were carried out. As mentioned before, mass transport can result from both advective-dispersive and diffusive processes. In the case of the *SC*, the assumed pressure gradient is directed outward (i.e., there is a higher fluid pressure at the base of the *SC *than at the surface), and this naturally results in fluid flow in an outward direction. As drugs are normally applied to the outer skin surface, source concentrations were placed at the top surface of the model (i.e., the skin surface). Therefore, in order for the drug to enter the skin, it must move against the direction of fluid flow (i.e., by diffusion alone).

We observe that for a specified continuously applied concentration of 10 mg/L at the top surface, the concentration increases at all observation points, and eventually levels off. We opted for this kind of boundary conditions so that we could simulate a transient process without a change in the source. If the source is removed, the simulated concentration would move as a pulse through the SC.

**Figure 8:**
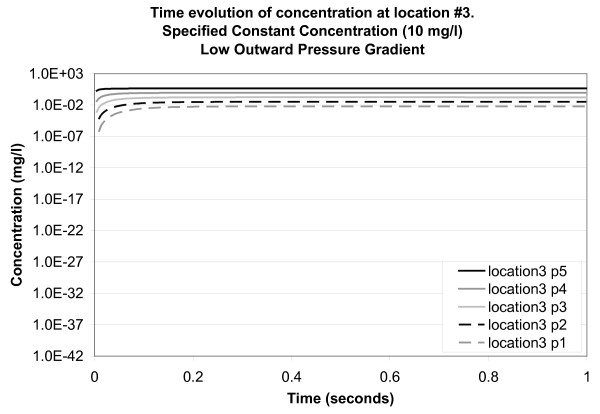
**Time evolution of concentration at location 3 for a specified constant concentration of 10 mg/l applied to the top surface of the model (low outward pressure gradient)**. Concentration is plotted on a logarithmic scale. Location points are listed in the legend in their order of depth in the model (5 at the top and 1 at the bottom).

Figure 8 shows the results for observation points solely within an intercellular channel (location 3) for the case of a low pressure gradient between the bottom and top surface of the *SC*. The concentration curves are of similar shape but, at any time point, their magnitudes reduce alongside distance to the top boundary of the model (i.e., point 5 has the highest concentrations and point 1 has the lowest). This is a predictable behaviour of breakthrough curves for continuous source problems.

**Figure 9:**
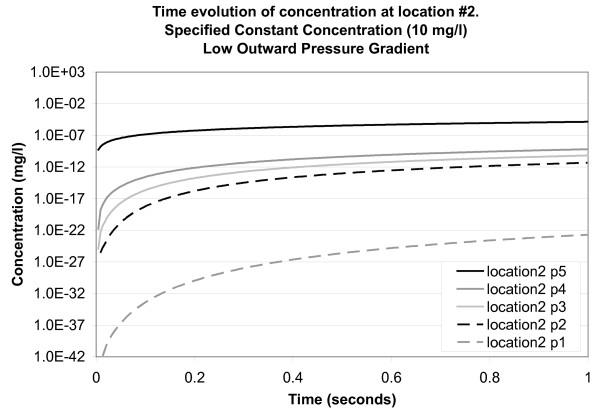
**Time evolution of concentration at location 2 for a specified constant concentration of 10 mg/l applied to the top surface of the model (low outward pressure gradient)**. Concentration is plotted on a logarithmic scale. Location points are listed in the legend in their order of depth in the model (5 at the top and 1 at the bottom).

The simulated concentrations for points within a corneocyte, followed by a wide intercellular channel, followed by a second corneocyte (at location 2) are shown in Figure 9. As illustrated in Figure 4, the location of observation point 5 is near the top and within a corneocyte; observation points 4, 3 and 2 are within an intercellular channel; and observation point 1 is within a lower corneocyte. Concentrations are observed to increase with time, although the maximum concentrations are considerably less than those at location 3, corroborating that mass transport is lessened by the presence of a corneocyte at the top of the model domain.

**Figure 10:**
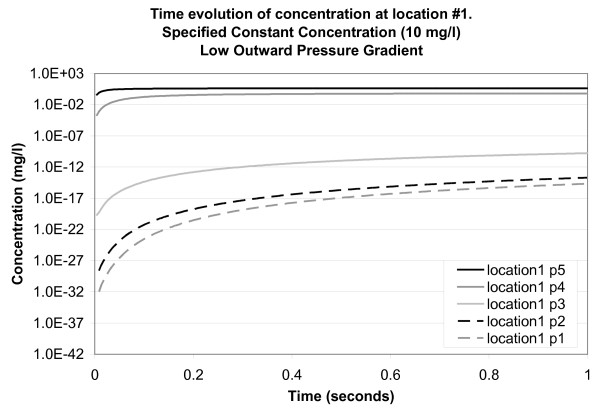
**Time evolution of concentration at location 1 for a specified constant concentration of 10 mg/l applied to the top surface of the model (low outward pressure gradient)**. Concentration is plotted on a logarithmic scale. Location points are listed in the legend in their order of depth in the model (5 at the top and 1 at the bottom).

Figure 10 shows the results for observation points within an intercellular channel, followed by a corneocyte, followed by a second intercellular channel (at location 1). In this case, the concentrations attained at the top two points (5 and 4) are significantly higher than that at point 3, which in turn is higher than both 1 and 2. Again, these results suggest that the presence of an intercellular channel enhances mass transport, while a corneocyte hinders it.

All together, our results show that the maximum rate of diffusion will be achieved through intercellular channels, and that, consistent with the flow regimes shown in Figure 6, transport will likely follow a tortuous pathway, selecting the route of maximum ease of transport. There is a direct correlation between lower permeability pathways within the material (i.e., corneocytes as opposed to intercellular channels) and a lower rate of mass transport. Specifically, we observed that concentrations are higher and breakthrough occurs at faster rates at locations dominated by intercellular channels. Mass transport is thus hindered by the presence of low permeability corneocytes. The results are generally consistent with what would be predicted on the basis of diffusion-dominated mass transport in the presence of a low opposing pressure gradient.

As would be intuitively expected, increasing the pressure gradient (outward) has the effect of reducing the amount of diffusion into the *SC *(results not shown). Concentration patterns under a high pressure gradient are similar to those described for the low pressure gradient, but the absolute concentrations at each point reach their maximum, albeit much lower values at earlier times. The largest difference is observed for location 3, which is situated within an intercellular channel, due to the higher upward flow of fluid countering the downward diffusion of mass. In the absence of strong flow, diffusion is uninhibited and downward transport occurs readily.

Experimental results for the transport of a model-drug (ketorolac tromethamine) into human skin *in vivo *[31] give a depth of penetration into the *SC *of approximately 10 *μ*m (the SC-viable epidermal junction) within a one hour period. Drug transport was facilitated by the use of both rigid and elastic vesicles, while depth profiles were measured using a tape-stripping method [32]. Their experimental design is likened to our case for a constant concentration applied to the skin surface (long period of exposure). In their experiment, roughly 2.4% (2.40 ± 0.69 *μ*g) of the amount of the model-drug applied to the skin surface (100 *μ*g) cumulatively reached the middle SC layers (corresponding to the base of our model domain). Considering the graph for point 1 (base of the model at 5 *μ*m), location 3 (intercellular channel) (Figure 8), the line of best fit (logarithmic) is C = 0.0035 log (time) - 0.0069. For an extrapolated time of 3600 sec (1 hour), the concentration is 0.02 mg/l (or 0.2% of the applied concentration).

Our simulations yield strikingly similar results as compared with the experimental findings, where the relative amount of ketorolac found in middle SC layers fluctuated between 0.1-0.3% (see Figure 4 of [31], tape-strips number 25-40). Given the uncertainty of some parameter values, such as exact pressure gradient, these results are entirely satisfactory. Moreover, the experimental results in [31] also suggested that the amount of model-drug in the deeper layers of the *SC *was found to be very little, as compared to what could be found on the skin surface. This is entirely consistent with our numerical results, which point to substantially reduced concentrations at depth within the *SC*.

**Figure 11:**
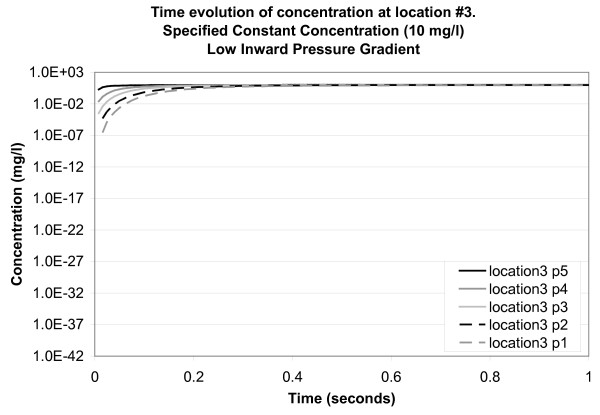
**Time evolution of concentration at location 3 for a specified constant concentration of 10 mg/l applied to the top surface of the model (low inward pressure gradient)**. Concentration is plotted on a logarithmic scale. Location points are listed in the legend in their order of depth in the model (5 at the top and 1 at the bottom).

Another consideration is the possibility that osmotic pressure, the result of a water gradient across the *SC*, is responsible for transport into and across the *SC *[33]. It has been observed that the water content in the deepest layers of the *SC *is low in comparison with the water content in its superficial or central regions [22]. In consideration of this possibility, one final simulation was conducted in which a low positive pressure gradient (inward) was applied across the model. Pressure results suggest that flow is directed downward along tortuous pathways around the corneocytes. Concentration results for location 3 (intercellular channel) point to enhanced transport, with concentrations attaining maximum concentrations above those obtained for the low outward pressure case (Figure 11). For comparison, concentrations at point 1 (5 *μ*m depth) stabilize at 9.45 mg/l (94.5% of the applied concentration). These results are much higher than the experimental results, which yielded 2.4% of the applied concentration at this depth, as stated above.

## Conclusions

Simulations undertaken in a standard porous medium fluid flow and transport model proved to be very useful for describing fluid flow pathlines within the heterogeneous skin structure as well as the behaviour of a solute placed at the top surface of the *SC*. Fluid pressure and velocity profiles are consistent with the behaviour that might be predicted based on permeability structure, regardless of the direction of the applied pressure gradient, and provide a good representation of flow through *SC *from both the physical and biological points of view. The fluid is shown to follow a tortuous path through the intercellular channels as defined by the presence of corneocytes.

The concentrations calculated at observation points, which were placed at discrete locations where the intercellular channels meet, demonstrate that drugs will theoretically penetrate the *SC*, regardless of whether the pressure gradient is directed outward or inward, and regardless of the magnitude of the pressure gradient. Simulations were initially conducted at a lower pressure gradient than those measured experimentally in order to observe the differences in mass diffusion processes recorded spatially within the *SC*. Increasing the magnitude of the pressure gradient to values determined experimentally has the effect of reducing the maximum concentrations attained at each point, particularly within the intercellular channels. Similarly, reversing the gradient such that there is an inward directed fluid flow results in enhanced transport. In all cases, diffusion results in mass transport of a solute placed at a specified continuous concentration at the top surface of the *SC*. The percentage of diffused drug within the *SC *in our model is consistent within reasonable ranges of the experimental results.

As could be expected from any macroscopic model, our results do not attempt to produce a full representation of molecular transport behaviour within the *SC*, as the *SC *was treated as an equivalent porous medium. More appropriate cellular-based conceptual models may exist. Nevertheless, the research does provide a reasonable conceptual model that appears to reproduce the expected behaviour of drug transport, which happens to be quite a physically reasonable and straightforward simple model.

Moreover, these simulation results scale adequately with the input data, which is consistent with the behaviour of the obtained family of solutions (cf. Appendix). Thus, it is expected that once the pressure gradient is accurately measured through experimentation, its input into the model would provide significant resemblance to the actual physical behaviour. Moreover, the model can be adapted for the assessment of particular drugs, considering their specific lipophilicity, size and solubility properties.

Among many topics for future research related to this work, explicit consideration of anisotropy within the intercellular channels should be examined, perhaps implying the creation of a multi- scaled porous media solver. Additionally, further extensive biological experimentation is needed to obtain accurate input data (e.g. pressure gradient), from which better estimates can be derived by using our proposed methodology.

## Appendix

Here, we want to provide an analytical 3D solution of a simple model that allows the comparison of cross-section head pressure contours with those obtained from our numerical simulations. From this comparison, we are able to address the head pressure contours as part of a family of solutions. Thus, quantitative arbitrary values of pressure always result in the same qualitative features. This is particularly important, since there is no known reference for the exact value of pressure within the SC.

**Figure 12:**
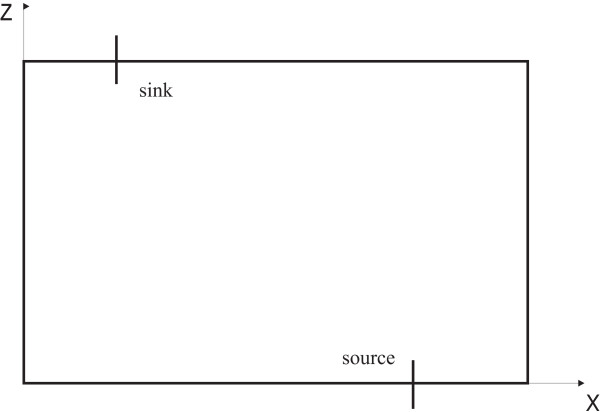
**Source and sink problem, in the two-dimensional reduction corresponding to a horizontal cross-section of the original flow problem**.

If we consider a two-dimensional reduction of the flow problem, corresponding to a horizontal cross-section of the original problem, it will contain a sink and a source in the x-z plane, mimicking the scattered pattern of the keratinocytes. A point source and a point sink are used to depict the limit where the intercellular channels are infinitely small, which could be considered true in comparison to the dimension of the coneocytes. The two-dimensional reduction is illustrated in Figure 12. By symmetry, we can consider an analogous solution for the y-z plane, and a solution to the original three-dimensional problem would then consist of the superposition of both periodic solutions. In this way, we can obtain the pressure contours in the x-y plane once the value of z is fixed.

Here, it should be noted that the usual way to describe a flow is through the expression ***u ***= ***u***($\vec{x}$, *t*), which indicates the flow velocity at any point $\vec{x}$, at any time *t*. In our case, since we are dealing with an incompressible and irrotational flow, the curl and divergence of the velocity should be zero. This is the reason why in our simplified case, constituted by a simply connected region, we can talk about a velocity potential *φ*, defined as ***u ***= ∇*φ *and a scalar stream function *ψ*($\vec{x}$, *y*) such that its level curves are always parallel to the velocity. The latter automatically satisfies the incompressibility condition, and since (***u ***·∇)*ψ *= 0 is we can see that the function *ψ *is constant along the fluid streamlines. We are able then to construct the so-called complex potential Φ = *φ *+ *iψ *which is an analytic function in the complex plane, as is generally handled in elementary fluid dynamics' books (such as [34]), where the imaginary part represents the flow contours.

With this idea in mind, and since we first want to represent the qualitative behaviour of the flow within our two-dimensional reduction in the y-z plane, let us consider the following BVP:

$$\begin{array}{c} \nabla^{2}\varphi =0,\;\text{with}\;\nabla \varphi \to 0\;\text{as x},\text{y}\to \infty ,\\ \varphi_{\text{y}}(\text{x},\text{y}=0)=\text{f}(\text{x})=\delta (\text{x}-\text{s}),\;\text{s}\in ℛ \end{array}$$

for a source located at a point *s *in the x-z plane, representing a generalized flow problem such as that illustrated by Figure 12.

Considering an arbitrary forcing for our boundary condition, the system's solution is

$$J(x,y)=-\frac{1}{\pi }\int_{-\infty }^{\infty }\int_{y}^{\infty }\frac{\bar{y}f(s)}{\left({\left(x-s\right)}^{2}+{\bar{y}}^{2}\right)}d\bar{y}\;\;ds$$

where

$$G(x,\;y)=\frac{y}{\pi ({\left(x-s\right)}^{2}+y^{2})}$$

is the Green's function for the 2D Laplace problem with inhomogeneous Dirichlet boundary conditions. Hence,

$$J(x,y)=\int_{-\infty }^{\infty }-\frac{1}{2\pi }Ln({\left(x-s\right)}^{2}+y^{2})f(s)\;\;ds$$

which has a complex potential of $\Phi =-\frac{1}{2\pi }Ln[{\left(z-s\right)}^{2}].$

The difficulty now lies in setting the boundary conditions properly, at a predefined position *y *= *L *For this purpose we will use the method of images (see [13] for derivation details), upon which we obtain

$$\Phi =-\frac{1}{\pi }Ln\left\{-\frac{2L}{\pi }\text{sinh}\left(\frac{(s-z)\pi }{2L}\right)\right\}.$$

This potential refers to the domain which contains a source as the 'bottom' boundary condition, while the 'top' boundary condition is zero-flux. We now need to consider a domain with a sink as a 'top' boundary condition, with a zero- flux as a 'bottom' boundary condition. In this way, we can superimpose the potentials, yielding that of our reduced 2D problem. To do this, we only need to consider the following BVP

$$\begin{array}{l} \nabla^{2}\varphi =0,\text{ with }\nabla \varphi \to 0\text{ as x},\text{ y}\to \infty ,\\ \;\varphi_{\text{y}}(\text{x},\text{y}=0)=\delta (\text{x}-\text{p}),\text{ p}\in ℛ. \end{array}$$

from which it directly follows that the potential should be

$$\Phi =\frac{1}{\pi }Ln\left\{-\frac{2L}{\pi }\text{sinh}\left(\frac{(p-z)\pi }{2L}\right)\right\}.$$

By symmetry, we can consider an analogous solution for the y-z plane, and a solution to the original 3D problem would consist of the superposition of both periodic solutions. In this way, we can obtain the pressure contours in the x-y plane once the value of z is fixed.

Through the addition of both x-z plane and y-z plane potentials on a periodic basis we are able to obtain a three-dimensional solution that depicts the head pressure contours within a layer of intercellular lipids. Such contours will be logically affected by the point sources and sinks along the horizontal boundary conditions, whereas the vertical boundary conditions are considered to be of the no-flux type, to represent the symmetry of the linear flow. We are now able to plot a cross-section of the solution, by fixing the value of z within the restrictions imposed by the dimensions of the intercellular channel.

We fixed the value of the height of the superimposed solutions to be at the middle of the intercellular channels. The result shown in Figure 7 represents the pressure contours at such level, where *z *= *i *·*L*/2.

## Competing interests

The authors declare that they have no competing interests.

## Authors' contributions

All authors participated in designing the study, and analyzing results. TML performed numerical simulations. TML and DA did the modelling. All authors read and approved the final manuscript.
